# Recent advances in pharmacotherapy of atrial fibrillation

**DOI:** 10.4103/0253-7613.56064

**Published:** 2009-08

**Authors:** J. Singh, J.S. Braich

**Affiliations:** Department of Pharmacology Pt. BD Sharma PGIMS, Rohtak-124 001, India

**Keywords:** Atrial fibrillation, antiarrhythmic drugs, angiotensin-converting enzyme inhibitors, angiotensin II type1 receptor blockers, normal sinus rhythm

## Abstract

Atrial fibrillation (AF) is the most common sustained arrhythmia associated with increased morbidity and mortality. Efficacy and safety of currently employed antiarrhythmic drugs (AADs) continue to be less optimal in AF. Development of newer AADs has recently been made possible through a greater understanding of electro-pathophysiology of AF. Highly specific drugs acting on atria are currently being explored, although there is little data available on effectiveness of atrial specific agents in maintaining sinus rhythm. Combining AADs and non-AADs such as angiotensin converting enzyme inhibitors (ACEIs) and angiotensin receptor blockers (ARBs) may increase effectiveness of AADs in patients with AF. Controlled clinical trials are required to precisely define the efficacy of single agents versus various combinations in maintaining sinus rhythm in patients with AF. This review describes some of the most promising therapeutic approaches that may overcome some of the limitations of drugs used at present for the management of AF.

## Introduction

Atrial fibrillation (AF) is a common and distressing arrhythmia with a prevalence of about one per cent in adult US population.[1] It is a major risk factor for stroke and mortality in North America.[2] The prevalence of AF increases with age such that eight per cent of people above 80 years of age have AF. The life time risk for development of AF is one in four for men and women aged 40 years and older. Projected data from population based studies in the US suggests that by 2050 a 2.5 to three fold upsurge in the number of adults with AF is expected.[1] However, even these projections may represent conservative estimates because of undiscovered silent AF. The high lifetime risk for AF and increased longevity underscore the important public health burden posed by it. Arrhythmias presently cost approximately one per cent of the health care budget in the US, UK and France. Because of the clinical importance and lack of highly satisfactory management approaches, AF is a subject of active clinical and research efforts. This review describes some of the most promising approaches to new therapies of AF.

## Pathophysiology of Atrial Fibrillation

AF has a multiplicity of causes ranging from genetic to degenerative but hypertension and heart failure are epidemiologically most prevalent conditions associated with it. Atrial structural remodeling creates a substrate for AF.[3] Renin- angiotensin-aldosterone system (RAAS) and Angiotensin II type1 receptor (AT_1_) have been implicated in atrial structural and electrical remodeling.[4,5] Experimental evidence suggests that RAAS activates mitogen activated protein kinase (MAPK) causing proliferation of fibroblast and hypertrophy of myocytes.[6] The resulting atrial scarring causes prolongation of refractory period and maintenance of arrhythmogenic substrate in the atrium resulting in AF.[7] Oxidative stress has recently been reported to be implicated in the pathophysiology of AF.[8] Additionally, stretch receptor antagonism, sodium-calcium exchanger blockade, late sodium channel inhibition and gap junction modulation have also been shown to be anti-arrhythmic mechanisms.[9]

## Pharmacotherapy of Atrial Fibrillation

In acute AF the precipitating cause which could be. fever, pneumonia, alcohol intoxication, thyrotoxicosis, pulmonary emboli, CHF or pericarditis should be treated first.[10] In severe cases electrical cardioversion is the treatment of choice, otherwise slowing of the ventricular rate should be the initial therapeutic goal by using β-blockers, calcium channel blockers (CCBs) or digitalis.[10] Conversion to a sinus rhythm may be attempted using AADs like quinidine, flecainide, ibutilide etc. Direct current (DC) cardioversion is the most effective method for restoring a sinus rhythm. To prevent recurrence of AF, quinidine, flecainide, propafenone, sotalol, dofetilide or amiodarone should be used.[10] Ablation therapy or MAZE procedure for cure of AF are other options.[10] At present both rate control and anticoagulation are recommended in elderly asymptomatic patients whereas in younger patients with symptomatic recurrent AF pulmonary vein isolation ablation is the treatment of choice.[9]

Conventional AADs are often used for conversion and long term suppression of AF in treatment, however, in limited use because of the modest efficacy, tolerability and potential for serious ventricular proarrhythmia and organ toxicity. The efficacy of AADs in maintaining normal sinus rhythm (NSR) is disappointing because most of the patients have recurrent AF despite treatment. Many trials with different AADs (CAST and SWORD) have shown increased mortality in the presence of structural heart disease.[11–12] The utility of pharmacotherapy to restore and maintain NSR has been questioned in recent trials which do not show a mortality benefit.[13–14] Therefore, there is still a need for more effective and safer drugs to promote NSR.

## Antiarrhythmic Drugs Under Investigation

### Atrial selective agents

An attractive prospect for pharmacotherapy of AF is the introduction of agents with selective affinity to ion channels specifically involved in atrial repolarization; the so called atrial repolarization delaying agents. Presently there are several AADs with this mode of action under preclinical and clinical development. The development of AADs with selective channel blocking profile has been made possible by a greater understanding of the pathophysiology of AF.[15] The finding that the ultra rapid delayed rectifier (I_Kur_) exists in the atria but not in ventricular tissue has offered the prospect of developing atrial selective drugs devoid of ventricular proarrhythmic toxicity. These atrial selective agents include I_Kur_ blockers, atrial selective sodium channel blockers, muscarinic (M_2)_ receptor blockers and five-HT_4_ receptor blockers.[16]

Vernaklant (RSD 1235) is in the most advanced phase of investigation and its (IV) formulation has recently been recommended for approval for pharmacological conversion of AF.[9] It is relatively atrial specific, the most completely studied atrial specific agent to date, AAD with mild voltage and frequency dependant sodium channel block as well as I_Kur_ and I_to_ block.[17] One phase II study with I.V. formulations has been published (CRAFT)[18] in which 56 patients with AF of 3-72 hours were randomized to RSD 1235 dose group or placebo. Thirty minutes after infusion AF was terminated in 61% of the patients in the higher dose drug group compared with five per cent in the placebo group. The drug did not prolong QT_c_ or QRS intervals or produce torsade de pointes (TdP). Adverse effects were rare and mild. Preliminary results have shown that 38% of patients receiving the drug were cardioverted versus four per cent in the control group. ACT-3 was a second pivotal trial that enrolled 276 patients and found a 41% conversion rate for the drug group versus four per cent for the placebo group. For recent onset AF, 52% of the patients converted versus four per cent in the placebo group. The drug was well tolerated and no TdP was observed. A separate trial on postoperative patients is also ongoing (ACT-2). When compared with the placebo I.V., Vernakalant appears to be both effective and safe for acute conversion in patients with AF.[18] However, its efficacy in patients with atrial flutter is uncertain. An oral formulation of Vernakalant is under development for long term maintenance of NSR following cardioversion.

#### AVE0118:

AVE0118 is an atrial selective potassium channel blocker inhibiting the ultra rapid component of the delayed rectifier (I_Kur_) which is present only in the atria and the transient outward current (I_to_) that is found more prominently in the atria. In animals, AVE0118 prolonged atrial refractoriness, notably in electrically remodeled atria, prolonged atrial wave length in a dose dependant fashion, reduced AF ability to induce and acutely converted persistent AF to sinus rhythm without altering the QT interval.[19]

#### AZD 7009:

AZD7009 is a mixed ion channel blocker, blocking the delayed rectifier potassium current (I_Kr_), sodium current (I_Na_) and I_Kur_, with eletrophysiological effects predominantly on the atrial tissue.[20] Both an I.V. formulation for acute conversion and oral formulation for long term treatment of AF are under phase II trials of development. It shows only a small effect on the ventricular effective refractory period (ERP) and QT interval.[21] In animal models AZD 7009 was effective for cardioversion of AF and atrial flutter. A human study had shown that 70% of the patients with AF receiving a high dose AZD 7009 were cardioverted at two hours with no cases of TdP.[22] However, the presence of mild prolongation of the QT interval, probably mediated by ventricular I_kr_ current, suggest some likely risk of TdP.

#### XEND 0101:

It blocks a single atrial specific membrane current.[9] Success of such agents depends critically on the atrial eletrophysiological selectivity, freedom from cardiac adverse effects and general safety.

### Amiodarone congeners

#### Dronedarone:

It is a non-iodinated amiodarone benzofurane with many antiarrhythmic properties corresponding to those of amiodarone.[23] A change in molecular structure leads to decline in adverse effects on thyroid and lung function while retaining the electro-physiological properties of amiodarone. In addition, the non specific sympatholytic (α and β-receptor blocking) and Ca^2+^ channel blocking actions of dronedarone confer a potential for ventricular rate control in AF recurrence. Dronedarone inhibits multiple ion channels in the heart including sodium, potassium and calcium currents (L-type), the rapid delayed rectifier and acetylcholine-activated potassium currents. In guinea pig ventricular myocytes dronaderone blocks sodium current (I_Na_), L-type Ca^2+^ current and several potassium currents (I_Kr_, I_Ks_, I_Ki_). The sodium channel blocking activity has also been demonstrated in human atrial myocytes.[23] In a rabbit model comparing long term oral administration of oral dronedarone with amiodarone, both the drugs increased action potential duration (APD) and effective refractory period (ERP) of the atrial tissue to a similar degree.

Dronedarone has been widely studied with several completed trials related to AF therapy. In a pooled analysis of results from two international phase III trials (EURIDIS and ADONIS) of dronedarone in maintenance of sinus rhythm in 1250 patients, with either paroxysmal (70%) or persistent AF, the first year data showed that compared with placebo the time of AF recurrences was 2.3-2.7 times longer after treatment with dronedarone 400 mg twice daily. Safety data were promising but patients with a heart failure were excluded from the trials.[24] A phase II study, DAFNE, enrolled patients undergoing cardioversion for AF with primary end point as time to AF recurrence; and a significant increase in time before AF recurrence was seen with dronedarone, in dose 800 mg/d, group (60 days versus five days in placebo group). Higher doses were not effective. At a dose 800 mg/d the QT interval was not prolonged and there was no evidence of ocular, pulmonary or thyroid toxicity.[25] A multinational study ATHENA is being conducted in over more than 4000 high risk patients with AF for further data on efficacy and safety of dronaderone; with the primary end point all cause mortality combined with cardiovascular hospitalization.[26] Additional studies are being conducted to evaluate use of dronedarone in patients with implantable cardioverter defibrillators.

## Celivarone (SSR 149744C)

It is another non-iodinated amiodarone derivative undergoing phase II human trials. It exhibits eletro-physiological and hemodynamic properties characteristic of dronedarone.[27]

### 

#### ATI-2042:

In this congener, iodination has been retained in phase II trials to assess the ability of the compound to suppress AF in patients with pacemakers. There is much evidence that amiodarone is a desirable model for development of antiarrhythmic compounds.[16]

#### Ranolazine:

There is increasing data indicating that amiodarone and its congeners i.e. dronedarone and ranolazine have the potential to variably increase ventricular repolarization by ionic mechanisms such that they are unlikely to provoke early depolarization produced by I_Kr_ blockers and thus have a lower risk of inducing TdP.[16]

### Other antiarrhythmic drugs

#### Azimilide:

It is a selective Class-III AAD that blocks both rapid (I_Kr_) and slow (I_Ks_) components of the delayed rectifier potassium current.[28] It prolongs cardiac APD and refractory period. Its long half life (up to four days) allows one daily dosing and limits major fluctuations in blood concentration. Several randomized, placebo controlled clinical trials have demonstrated the efficacy of azimilide in prolonging symptom free interval in patients with AF or atrial flutter.[29] In meta-analysis, azimilide in dose 10 mg and 125 mg demonstrated greater efficacy when compared with placebo in prolonging the symptomatic arrhythmia free interval.[30] However the effects of the drug with respect to maintaining sinus rhythm remain unclear.

#### Tedisamil:

An antianginal agent possessing multiple ion channel effects including blockade of transient outward current I_to_, in addition to I_Kr_, I_Ks_, I_Kur_, I_K-ATP_ and even I_Na_[31] also causes reverse, rate dependant QT interval prolongation. In a multicenter, double blind, randomized placebo controlled study in 175 patients with symptomatic recent onset AF or atrial flutter, 41-51% of patients receiving tedisamil (0.4 or 0.6 mg/kg I.V.) converted to sinus rhythm in an average time of 35 minutes with two instances of (1.8%) of possible proarrhythmia.[32]

#### Rotigaptide (ZP 123):

It is a specific gap-junction-facilitating drug. Gap junctions are specialized pores that coordinate cell-to-cell transmission of electrical impulses essential for synchronized contraction.[33] Gap-junction modulation may present a novel therapeutic target in some forms of currently being studied in a phase II trial on rotigaptide. Rotigaptide has been demonstrated to reduce AF vulnerability in a canine model of chronic mitral regurgitation but not in a ventricular tachy-pacing model that results in atrial fibrosis. It also reduces AF in a canine model of atrial ischemia.[34] Identification of those patients who will benefit from improving gap-junction conduction will require further study.

#### Serotonin (5-HT_4_) receptor antagonists:

According to recent data 5-HT_4_ receptor antagonists could be promising drugs in patients with AF as infusion of serotonin induces sinus tachycardia and other atrial tachyarrhythmias including AF. Atrial-specific 5-HT_4_ receptor subtypes have been observed and can be targeted pharmacologically without potentially inducing ventricular side effects. Serotonin 5-HT_4_ receptor antagonists, currently in development, include RS-100302, SB-207266 and CVT-150.[35]

#### Angiotesin converting enzyme inhibitors and angiotensin receptor blockers:

ACEIs or ARBs may prevent AF in some patients. Angiotensin system inhibition appears to protect against AF in patients with hypertension, LV hypertrophy, post MI with LV dysfunction and chronic CHF. The effect is clearest in patients with LV systolic dysfunction and CHF.[36] Kishley demonstrated that administration of ACEIs or ARBs was associated with overall 18% risk reduction in new onset of AF across the trials and 43% risk reduction in patients with heart failure.[37] Kalus demonstrated that administration of ACEIs or ARBs for at least three months prior to cardiothoracic surgery was associated with reduction in postoperative AF.[38] There are several potential mechanisms by which these drugs may reduce AF. these include direct modulation of ion channels, hemodynamic improvement, electrical structural remodeling, antifibrotic, anti-inflammatory, antiplatelet, antiproliferative and reducing Ca^2+^ influx and oxidative stress etc.[39] These drugs appear to have promising use following cardioversion, but require further study.

#### Statins:

Statins appear to have a role in prevention of AF. In a randomized trial (ARMYDA-3) enrolling 200 statin-naïve patients to atorvastatin or placebo before cardiac surgery, atorvastatin treated group showed 61% reduction in risk of postoperative AF.[40] Bhavnani et. al demonstrated that among a cohort with implantable cardioverter defibrillators (ICDs) at high risk of cardiac arrhythmias, statin therapy was associated with reduction in AF and atrial flutter (AF/AFL); adjusted hazard ratio was less for the development of AF/AFL with shock therapy than without shock therapy.[41]

#### Fish oil (Omega-3PUFA):

There is an inverse epidemiological association between fish consumption and incidence of AF, with a 31% reduction in AF for elderly US adults who eat fish more than five times a week compared those with less than once a month.[42] This protective effect was not found by another group using the Rotterdam data base.[43] One trial randomized 160 patients to 2g/d poly-unsaturated fatty acids or placebo before coronary artery bypass graft. There was 54% reduction in post-operative AF in drug group.[44] There is now a large ongoing prospective trial of omega-3 PUFA in patients with paroxysmal or persistent AF.

Fibrosis is a feature of mechanical remodeling in long standing AF, particularly when associated with heart failure. Pirfenidone, an antifibrotic drug, significantly reduced arrhythmogenic atrial remodeling and AF vulnerability in a dog model of heart failure.[45] Ozaydin *et al*. demonstrated that N-acetycysteine treatment decreased incidence of postoperative AF in 115 patients that undervent coronary artery bypass or valve surgery.[8] Mutations in the connexin-40 protein have recently been identified in sporadic cases of human AF and gap junctions are an emerging target for AF.[46]

## Combined Therapy in Atrial Fibrillation Management

Recently it has been shown that sinus rhythm achieved after conversion of AF may be prolonged by certain non-antiarrhythmic drugs such as ACEIs, corticosteroids, aldosterone antagonists, statins and omega 3-PUFA. Rate of AF recurrence was lower when amiodarone combined with ACEI than with amiodarone alone.[47] Madrid *et al.* compared two groups of patients with one episode of AF for more than seven days; after conversion one group was treated with amiodarone (400mg/d) and second group with amidarone plus irbesartan (150-300 mg/d). Of the total 186 patients for follow up time 245 days, patients treated with irbesartan plus amiodarone had a greater probability of remaining free of AF (79.52% vs. 55.91%, p = 0.007).[48] Pedersen *et al.* investigated the effect of trandolapril on the incidence of AF, concluding that significantly more patients on placebo developed AF (n = 42; 5.3%) than those on trandolapril (n = 22; 2.8%). The data, though promising has not been derived from prospective blind and control clinical trials. It has been emphasized that both β-blockers and ACEIs exert significant effect on long term maintenance of sinus rhythm in patients with AF.[49] β-blockers and ACEIs can be combined with long term amiodarone therapy to prolong the duration of sinus rhythm after in a majority of patients with AF.

## Conclusion

AF remains a difficult clinical condition for the cardiologist as current AADs are unsatisfactory with limited efficacy and serious side effects. Atrial selective agents such as Vernakalant, AVEO 118 and AZD 7009 are promising. Dronedarone, with less adverse effects is useful particularly for long term treatment AF and may become first line agent for patients with AF. Currently, amiodarone is the first line agent for maintaining NSR in patients with heart failure or significant LV hypertrophy. Drugs that modulate fibrosis and connexins are undergoing testing in animal models and may provide new targets for treatment of AF. Statins and omega3-PUFA appear to be effective in preventing AF in some of the patients, but further trials are required to establish the role. Antifibrotic effects of angiotensin system antagonism and gene therapy approaches might add to AF therapeutic options in future. Results of still pending trials, novel compound development and evolution of ablation procedures will determine the treatment of AF in the near future.
